# Sequence Divergence of the Enniatin Synthase Gene in Relation to Production of Beauvericin and Enniatins in *Fusarium* Species 

**DOI:** 10.3390/toxins5030537

**Published:** 2013-03-13

**Authors:** Łukasz Stępień, Agnieszka Waśkiewicz

**Affiliations:** 1 Institute of Plant Genetics, Polish Academy of Sciences, Strzeszyńska 34, Poznań 60-479, Poland; 2 Department of Chemistry, Poznan University of Life Sciences, Wojska Polskiego 75, Poznań 60-625, Poland; E-Mail: agat@up.poznan.pl

**Keywords:** beauvericin, cyclic peptides, DNA markers, enniatins, *Fusarium* spp., phylogeny

## Abstract

Beauvericin (BEA) and enniatins (ENNs) are cyclic peptide mycotoxins produced by a wide range of fungal species, including pathogenic *Fusaria.* Amounts of BEA and ENNs were quantified in individual rice cultures of 58 *Fusarium* strains belonging to 20 species, originating from different host plant species and different geographical localities. The species identification of all strains was done on the basis of the *tef-*1α gene sequence. The main aim of this study was to analyze the variability of the *esyn1* gene encoding the enniatin synthase, the essential enzyme of this metabolic pathway, among the BEA- and ENNs-producing genotypes. The phylogenetic analysis based on the partial sequence of the *esyn1* gene clearly discriminates species producing exclusively BEA from those synthesizing mainly enniatin analogues.

## 1. Introduction

The fact that *Fusaria* are one of the most versatile mycotoxin producers is caused both by the wide range of species and the abilities of simultaneous biosynthesis of multiple metabolites from different metabolic pathways. The coincidence of trichothecenes and zearalenone produced by *F. graminearum* and *F. culmorum*, as well as fumonisins, beauvericin and moniliformin by *F. proliferatum* are primary examples [1,2]. The versatility of the *Fusaria* is frequently reflected by contamination of food and feed products with multiple mycotoxins [3,4,5].

**Figure 1:**
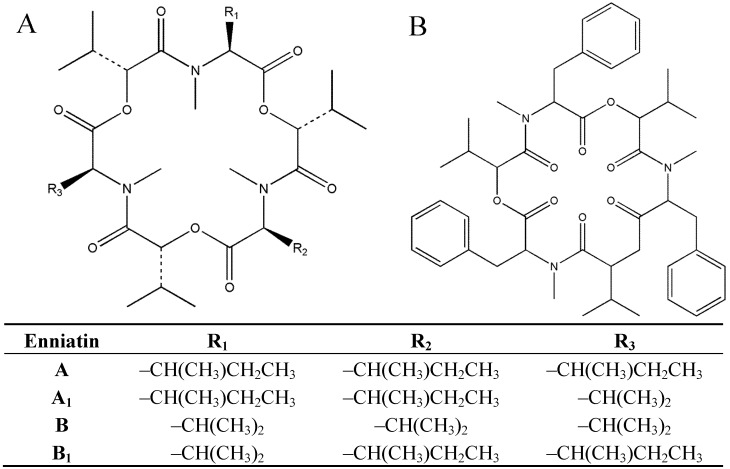
Chemical structures of (**A**) enniatins and (**B**) beauvericin.

Beauvericin (BEA), as well as a number of enniatin analogues: A, A_1_, A_2_, B, B_1_, B_2_ and B_4_ (ENNs)—belong to the cyclic hexadepsipeptide mycotoxins synthesized by numerous pathogenic fungi that are considered as a group of the emerging *Fusarium* mycotoxins. The spectral characteristics of those metabolites were revealed [6], and their molecular structures and toxicities were summarized by Jestoi [7]. In beauvericin, the three amino acid residues are aromatic *N*-methyl-phenylalanines, whereas in the enniatins of type A and B, the amino acid residues are aliphatic *N*-methyl-valine or -isoleucine or mixtures of these two (Figure 1; [8]). BEA and ENNs can be produced efficiently by strains of numerous *Fusarium* species *in vitro* and *in planta* [9,10,11,12,13,14,15].

The extent of human, animal and plant exposure to these mycotoxins has not been well established. The primary toxic action of BEA and ENNs is related to their ionophoric properties that disturb the physiological ionic balance and pH by forming dimeric structures transporting monovalent ions across the cell membranes [16,17]. Beauvericin is toxic to several human cell lines and can induce apoptosis and DNA fragmentation [18,19,20]. Moreover, in experimental animals, BEA exerted a negative inotropic effect (decrease in cardiac contraction strength), as well as a negative chronotropic effect (decrease in frequency of cardiac spontaneous beating activity) [21]. Investigation of the *Fusarium* genus showed that various species produced BEA, including some strains of *F. oxysporum* isolated from maize, pineapple and melon [22,23], *F. subglutinans* isolated from maize ears [24], *F. verticillioides* from pineapple [25] and *F. proliferatum* from maize, garlic and asparagus [26].

Enniatins are of high interest, because of their wide range of biological activity [27,28]. This bioactivity has long been assumed to be associated with their ionophoric properties [29]. ENNs inhibit the enzyme, acyl-CoA:cholesterol acyl transferase (ACAT) [30]. In cancer-related studies, enniatins were found to induce apoptosis and disrupt extracellular-regulated protein kinase associated with cell proliferation [31,32]. They are also known as phytotoxins and are associated with plant diseases characterized by wilt and necrosis [33].

The enniatin synthase gene (*esyn1*) has been proven to be the crucial one in the metabolic pathway of enniatin synthesis [34,35]. Moreover, a genomic locus containing a beauvericin biosynthetic gene cluster in the entomopathogenic fungus, *Beauveria bassiana*, has been cloned. Consequently, significant sequence homologies to certain *Fusarium* enzymes were found [36]. Recently, the homologous cluster from *F. proliferatum* was sequenced, and the gene encoding ketoisovalerate reductase—an enzyme controlling the initial step of the pathway—was characterized [37]. 

Some *Fusarium* species (like *F. poae*) have been reported to produce enniatins and beauvericin simultaneously [38], which is well justified by the fact that both mycotoxins share a common metabolic pathway. The co-occurrence of ENNs and BEA in field samples infected by *Fusarium* spp. has been observed [19,39]. There is a strong possibility that BEA and ENNs producers can be differentiated on the basis of the *esyn1* sequence [12]. Similar approaches based on genes from respective clusters (*i.e.*, *TRI*, *ZEA* and *FUM*) have been successfully applied to detect and characterize the chemotypes and populations of the potential producers of trichothecenes, zearalenone and fumonisins [35,40,41,42,43,44]. Therefore, the main objective of the present study was to examine the relation between the sequence variability inside the *esyn1* gene and the composition of the toxic cyclic peptides synthesized. 

The specific aims of this work were: (i) to examine the amounts of enniatins and beauvericin produced by the strains of various *Fusarium* species, (ii) to compare the phylogenetic relationships among the species revealed by the *tef-*1α sequence analysis to those reconstructed on the basis of the enniatin synthase gene, and (iii) to analyze the sequence variants of the *esyn1* gene coding regions among the strains studied in relation to the ratio between BEA and ENNs synthesized.

## 2. Results and Discussion

### 2.1. Fusarium Species Identification

Fifty-eight *Fusarium* strains belonging to 20 species stored at the KF Collection, Institute of Plant Genetics, Polish Academy of Sciences, Poznań, Poland, were used in the study. They represented both soil saprophytes as well as plant pathogens originating from 15 host species (Table 1). Most of the crop species are agriculturally important, regardless of the climatic conditions. Thus, the cosmopolitism of *Fusarium* pathogens and their ability to colonize a wide range of hosts is consistent with the isolates used in this study.

**Figure 2:**
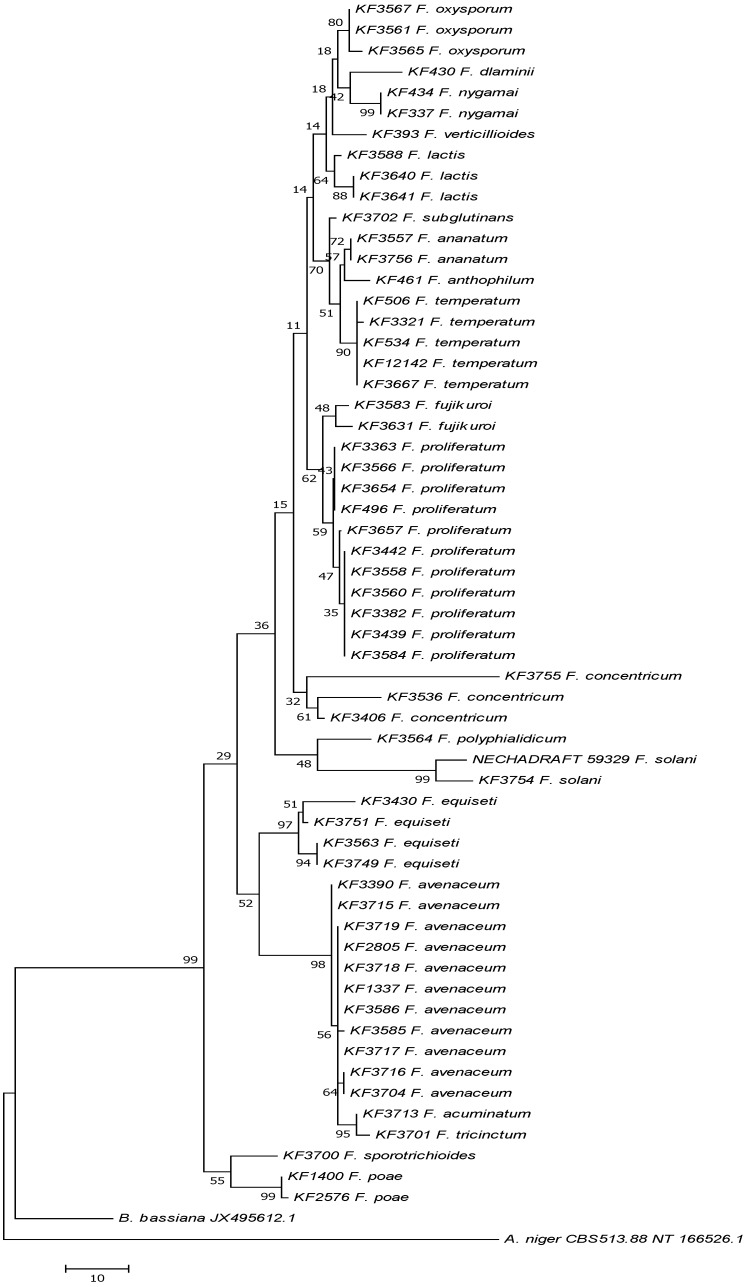
The most parsimonious tree for 57 *Fusarium* strains of 20 species used in the study, based on the translation elongation factor 1α (*tef*-1α) sequences. *F. solani*, *B. bassiana* (GenBank: JX495612.1) and *A. niger* (GenBank Acc. NT166526.1) sequences were included as the reference, as well as for outgrouping. The maximum parsimony approach and bootstrap test (1000 replicates) were applied.

Species identification was confirmed by the analysis based on the BLASTn comparison of the *tef-*1α gene sequences with the accessions deposited in the NCBI GenBank database. One strain of *F. sporotrichioides* (KF 3713) failed to amplify the marker fragment of the *tef-*1α gene. In this case, β-tubulin sequencing was the basis of the species identification (results not shown). All species were proven to have been identified correctly, showing the highest similarity level to the GenBank accessions belonging to the corresponding taxa, though strains of species, like *F. fujikuroi*, *F. proliferatum* and *F. temperatum*, appeared to be very closely related. Based on the obtained *tef-*1α sequences, a maximum parsimony dendrogram was calculated in order to show the level of the divergence among the genotypes. Additionally, the sequences of *F. solani*, *Aspergillus niger* and *Beauveria bassiana* were included in the analysis (Figure 2).

**Table 1:** *Fusarium* isolates used in the study, host plant species, year of isolation and geographical origin.

Strain	Species	Host	Year of isolation	Origin
KF 3713	*F. acuminatum*	*Pisum sativum*	2012	Poland
KF 3557	*F. ananatum*	*Ananas comosus*	2011	Costa Rica
KF 3756	*F. ananatum*	*Ananas comosus*	2011	Costa Rica
KF 461	*F. anthophilum*	*Plantago lanceolata*		USA
KF 1337	*F. avenaceum*	*Triticum aestivum*	1987	Poland
KF 3585	*F. avenaceum*	*Allium cepa*		Italy
KF 3586	*F. avenaceum*	*Lycopersicon esculentum*	2011	Poland
KF 3719	*F. avenaceum*	*Pisum sativum*	2012	Poland
KF 3718	*F. avenaceum*	*Pisum sativum*	2012	Poland
KF 3717	*F. avenaceum*	*Pisum sativum*	2012	Poland
KF 2805	*F. avenaceum*	*Triticum aestivum*	2009	Poland
KF 3704	*F. avenaceum*	*Zea mays*	2011	Poland
KF 3716	*F. avenaceum*	*Pisum sativum*	2012	Poland
KF 3390	*F. avenaceum*	*Zea mays*	2009	Poland
KF 3715	*F. avenaceum*	*Pisum sativum*	2012	Poland
KF 3755	*F. concentricum*	*Ananas comosus*	2011	Costa Rica
KF 3536	*F. concentricum*	*Ananas comosus*	2010	Costa Rica
KF 3406	*F. concentricum*	*Ananas comosus*	2009	Costa Rica
KF 430	*F. dlaminii*	*soil*		RSA
KF 3751	*F. equiseti*	*Lycopersicon esculentum*	2012	Poland
KF 3749	*F. equiseti*	*Lycopersicon esculentum*	2012	Poland
KF 3430	*F. equiseti*	*Musa sapientum*	2010	Ecuador
KF 3563	*F. equiseti*	*Asparagus officinalis*	2011	Poland
KF 3631	*F. fujikuroi*	*Oryza sativa*	2011	Thailand
KF 3583	*F. fujikuroi*	*Oryza sativa*	2011	Italy
KF 3588	*F. lactis*	*Capsicum annuum*	2011	Poland
KF 3641	*F. lactis*	*Capsicum annuum*	2011	Poland
KF 3640	*F. lactis*	*Capsicum annuum*	2011	Poland
KF 337	*F. nygamai*	*Cajanus indicus*		India
KF 434	*F. nygamai*	*soil*		Australia
KF 3561	*F. oxysporum*	*Allium sativum*	2011	Poland
KF 3567	*F. oxysporum*	*Allium sativum*	2011	Poland
KF 3565	*F. oxysporum*	*Asparagus officinalis*	2011	Poland
KF 1400	*F. poae*	*Zea mays*	1990	Poland
KF 2576	*F. poae*	*Zea mays*	1999	Poland
KF 3564	*F. polyphialidicum*	*Ananas comosus*	2011	Costa Rica
KF 3560	*F. proliferatum*	*Rheum rhabarbarum*	2011	Poland
KF 3442	*F. proliferatum*	*Zea mays*	2006	Poland
KF 3657	*F. proliferatum*	*Ananas comosus*	2011	Indonesia
KF 3566	*F. proliferatum*	*Oryza sativa*	2011	Thailand
KF 3439	*F. proliferatum*	*Ananas comosus*	2010	Ecuador
KF 496	*F. proliferatum*	*Zea mays*	1983	Italy
KF 3363	*F. proliferatum*	*Allium sativum*	2009	Poland
KF 3382	*F. proliferatum*	*Ananas comosus*	2009	Hawaii
KF 3584	*F. proliferatum*	*Oryza sativa*	2011	Thailand
KF 3558	*F. proliferatum*	*Asparagus officinalis*	2011	Poland
KF 3654	*F. proliferatum*	*Zea mays*	2011	Poland
KF 3754	*F. solani*	*Lycopersicon esculentum*	2012	Poland
KF 3700	*F. sporotrichioides*	*Asparagus officinalis*	2012	Poland
KF 3728	*F. sporotrichioides*	*Pisum sativum*	2012	Poland
KF 3702	*F. subglutinans*	*Cambria sp.*	2012	Poland
KF 534	*F. temperatum*	*Zea mays*	1985	Poland
KF 506	*F. temperatum*	*Zea mays*	1985	Poland
KF 1214,2	*F. temperatum*	*Zea mays*	1987	Poland
KF 3321	*F. temperatum*	*Ananas comosus*	2008	Costa Rica
KF 3667	*F. temperatum*	*Zea mays*		Belgium
KF 3701	*F. tricinctum*	*Asparagus officinalis*	2012	Poland
KF 393	*F. verticillioides*	*Zea mays*		USA

### 2.2. Method Validation and Recovery

Table 2 summarizes the linearity, limits of detection (LOD) and limits of quantification (LOQ) for enniatins and beauvericin. The linearity of the standard curves at three determinations of six concentration levels was reliable between 0.9976 and 0.9995. LOQ was calculated as three-fold LOD.

**Table 2:** Linearity (R^2^), limit of detection (LOD) and quantification (LOQ) (ng g^−1^) for mycotoxins.

Mycotoxin	*R*^2^ ^a^	LOD ^b^ (ng g^−1^)	LOQ ^c^ (ng g^−1^)
Enniatin A	0.9991	10.0	30.0
Enniatin A_1_	0.9976	10.0	30.0
Enniatin B	0.9993	8.0	24.0
Enniatin B_1_	0.9991	8.0	24.0
Beauvericin	0.9995	15.0	45.0

^a^ Regression coefficient; ^b^ Limit of detection (LOD); ^c^ Limit of quantification (LOQ).

Recovery rates and standard deviations were calculated at three concentration levels for black rice samples (Table 3). When analyzed mycotoxins were added to black rice within the range of concentrations from 5 to 60 ng g^−1^, the recovery rates were 92.8%–95.1%, 85.7%–90.2%, 94.3%–97.1%, 89.8%–91.4% and 98.3%–101.4% for ENNs: A, A_1_, B, B_1_ and BEA, respectively.

**Table 3:** Recovery of enniatins and beauvericin added to rice samples.

Mycotoxin	Quantity added (ng g^−1^)	Mean recovery (%)	Relative standard deviation (%)
Enniatin A	5	92.8	5.5
	20	95.1	4.8
	60	94.7	5.9
Enniatin A_1_	5	88.6	6.7
	20	90.2	5.9
	60	85.7	7.3
Enniatin B	5	95.2	6.8
	20	97.1	5.5
	60	94.3	6.3
Enniatin B_1_	5	89.8	4.3
	20	91.4	5.0
	60	91.2	6.8
Beauvericin	5	99.6	5.6
	20	101.4	4.9
	60	98.3	5.4

### 2.3. *In Vitro* Mycotoxin Biosynthesis

Amounts of enniatins and beauvericin produced by the strains of 20 *Fusarium* species were measured using the HPLC method. The results are summarized in Table 4.

**Table 4:** Mean concentration levels with standard deviations of beauvericin and enniatins (A, A_1_, B, B_1_) (in μg g^−1^) produced *in vitro* by *Fusarium* strains of 20 species.

Strain	Species	BEA (μg g^−1^)	ENN A (μg g^−1^)	ENN A_1_ (μg g^−1^)	ENN B (μg g^−1^)	ENN B_1_ (μg g^−1^)
KF 3713	*F. acuminatum*	5.31 ± 0.77	19.62 ± 2.81	26.92 ± 1.97	90.89 ± 7.54	31.49 ± 5.90
KF 3557	*F. ananatum*	27.68 ± 1.88	6.94 ± 0.42	ND	8.81 ± 0.73	27.60 ± 2.25
KF 3756	*F. ananatum*	39.57 ± 2.63	11.18 ± 1.29	ND	ND	27.07 ± 1.92
KF 461	*F. anthophilum*	141.97 ± 10.67	7.11 ± 0.53	ND	6.17 ± 0.63	12.14 ± 0.85
KF 1337	*F. avenaceum*	ND	34.55 ± 4.18	71.90 ± 10.43	895.46 ± 55.48	452.46 ± 30.33
KF 3718	*F. avenaceum*	ND	ND	ND	7.97 ± 0.54	15.99 ± 0.95
KF 3717	*F. avenaceum*	ND	6.09 ± 0.88	5.65 ± 2.33	6.71 ± 0.72	11.46 ± 0.93
KF 2805	*F. avenaceum*	ND	ND	25.56 ± 4.19	40.09 ± 2.21	41.49 ± 5.32
KF 3704	*F. avenaceum*	ND	ND	ND	10.80 ± 0.87	117.77 ± 9.86
KF 3716	*F. avenaceum*	ND	12.67 ± 2.06	ND	5.99 ± 0.51	18.15 ± 2.00
KF 3390	*F. avenaceum*	ND	29.12 ± 3.21	32.40 ± 2.08	255.08 ± 18.76	138.15 ± 10.14
KF 3715	*F. avenaceum*	ND	8.99 ± 1.42	ND	194.90 ± 20.22	27.21 ± 2.17
KF 3755	*F. concentricum*	312.20 ± 28.09	11.40 ± 1.88	8.69 ± 0.75	17.33 ± 1.09	18.17 ± 1.44
KF 3536	*F. concentricum*	1928.83 ± 60.77	ND	41.36 ± 5.33	39.44 ± 1.88	28.58 ± 2.09
KF 3406	*F. concentricum*	0.42 ± 0.02	ND	ND	ND	6.98 ± 0.54
KF 430	*F. dlaminii*	ND	6.92 ± 5.41	6.28 ± 0.71	ND	7.61 ± 1.13
KF 3751	*F. equiseti*	ND	ND	6.94 ± 1.19	ND	7.66 ± 4.62
KF 3749	*F. equiseti*	ND	39.27 ± 2.14	38.18 ± 2.01	ND	29.22 ± 3.22
KF 3430	*F. equiseti*	ND	31.17 ± 2.81	32.15 ± 1.42	32.98 ± 2.63	41.22 ± 2.31
KF 3563	*F. equiseti*	ND	43.47 ± 3.76	36.81 ± 2.88	29.18 ± 2.14	30.39 ± 1.54
KF 3631	*F. fujikuroi*	428.09 ± 23.61	ND	ND	ND	ND
KF 3583	*F. fujikuroi*	5.60 ± 0.27	ND	ND	ND	ND
KF 3588	*F. lactis*	ND	ND	10.57 ± 1.02	9.59 ± 1.07	32.43 ± 4.55
KF 3641	*F. lactis*	ND	30.97 ± 1.97	26.94 ± 4.61	ND	ND
KF 3640	*F. lactis*	ND	ND	30.53 ± 3.32	27.63 ± 1.88	ND
KF 337	*F. nygamai*	22.86 ± 2.66	10.45 ± 1.58	ND	9.50 ± 0.84	ND
KF 434	*F. nygamai*	18.33 ± 1.09	8.15 ± 1.03	5.21 ± 0.32	8.69 ± 1.05	ND
KF 3561	*F. oxysporum*	46.12 ± 5.87	ND	ND	ND	ND
KF 3567	*F. oxysporum*	80.03 ± 10.23	ND	6.42 ± 0.66	8.25 ± 1.11	7.28 ± 0.32
KF 3565	*F. oxysporum*	20.06 ± 2.66	ND	ND	ND	ND
KF 1400	*F. poae*	394.67 ± 25.87	ND	ND	ND	ND
KF 2576	*F. poae*	37.53 ± 4.87	34.31 ± 2.57	26.89 ± 2.18	28.71 ± 3.45	ND
KF 3564	*F. polyphialidicum*	ND	ND	ND	ND	ND
KF 3560	*F. proliferatum*	149.67 ± 10.33	ND	ND	ND	ND
KF 3442	*F. proliferatum*	52.01 ± 3.68	ND	ND	ND	ND
KF 3657	*F. proliferatum*	74.08 ± 5.14	ND	ND	ND	ND
KF 3566	*F. proliferatum*	90.85 ± 10.21	ND	ND	ND	ND
KF 3439	*F. proliferatum*	8.61 ± 0.99	ND	ND	ND	ND
KF 496	*F. proliferatum*	ND	ND	5.48 ± 0.77	9.61 ± 1.06	12.89 ± 2.11
KF 3363	*F. proliferatum*	45.13 ± 5.56	ND	ND	ND	ND
KF 3382	*F. proliferatum*	3.39 ± 0.35	ND	ND	ND	ND
KF 3584	*F. proliferatum*	291.87 ± 32.65	ND	6.39 ± 0.32	12.92 ± 2.17	19.64 ± 1.18
KF 3558	*F. proliferatum*	78.07 ± 9.47	ND	5.82 ± 0.65	7.91 ± 0.92	10.27 ± 1.32
KF 3654	*F. proliferatum*	76.39 ± 10.15	ND	ND	8.26 ± 0.31	6.84 ± 0.87
KF 3754	*F. solani*	ND	ND	ND	ND	ND
KF 3700	*F. sporotrichioides*	8.33 ± 1.11	ND	ND	ND	ND
KF 3728	*F. sporotrichioides*	5.13 ± 0.37	12.67 ± 3.76	ND	5.99 ± 0.76	18.15 ± 3.06
KF 3702	*F. subglutinans*	13.05 ± 2.09	20.33 ± 2.88	ND	10.74 ± 2.08	29.50 ± 4.17
KF 534	*F. temperatum*	18.22 ± 3.44	17.65 ± 1.05	ND	ND	ND
KF 506	*F. temperatum*	17.47 ± 2.21	ND	ND	15.17 ± 2.22	9.88 ± 1.22
KF 1214,2	*F. temperatum*	4.47 ± 0.59	ND	ND	6.83 ± 1.21	8.10 ± 0.93
KF 3321	*F. temperatum*	290.97 ± 18.62	27.79 ± 3.46	34.39 ± 2.80	39.20 ± 5.07	29.21 ± 2.80
KF 3667	*F. temperatum*	11.40 ± 0.98	ND	ND	ND	ND
KF 3701	*F. tricinctum*	1.09 ± 0.29	ND	30.49 ± 4.15	68.55 ± 5.42	21.74 ± 2.56
KF 393	*F. verticillioides*	2.34 ± 0.53	ND	ND	8.75 ± 1.85	12.43 ± 3.41

ND—not detected.

Not surprisingly, the most efficient ENNs producers were found among *F. avenaceum* strains, and BEA was synthesized mostly by *F. concentricum*, *F. oxysporum*, *F. proliferatum*, *F. fujikuroi* and *F. poae* strains. There were only a few species producing exclusively BEA (*F. fujikuroi*, *F. proliferatum*, *F. oxysporum*) and ENNs (*F. avenaceum*, *F. equiseti*, *F. lactis*). The majority of the strains synthesized a mixture of BEA and ENNs (Table 4). Only *F. polyphialidicum* and *F. solani* did not make these mycotoxins. One of the most interesting strains was *F. temperatum* KF 3321, which produced remarkable amounts of BEA and ENNs, although BEA was about eight-fold lower than in the *F. concentricum* isolate, KF 3536. 

### 2.4. Enniatin Synthase (esyn1) Gene Divergence

**Figure 3:**
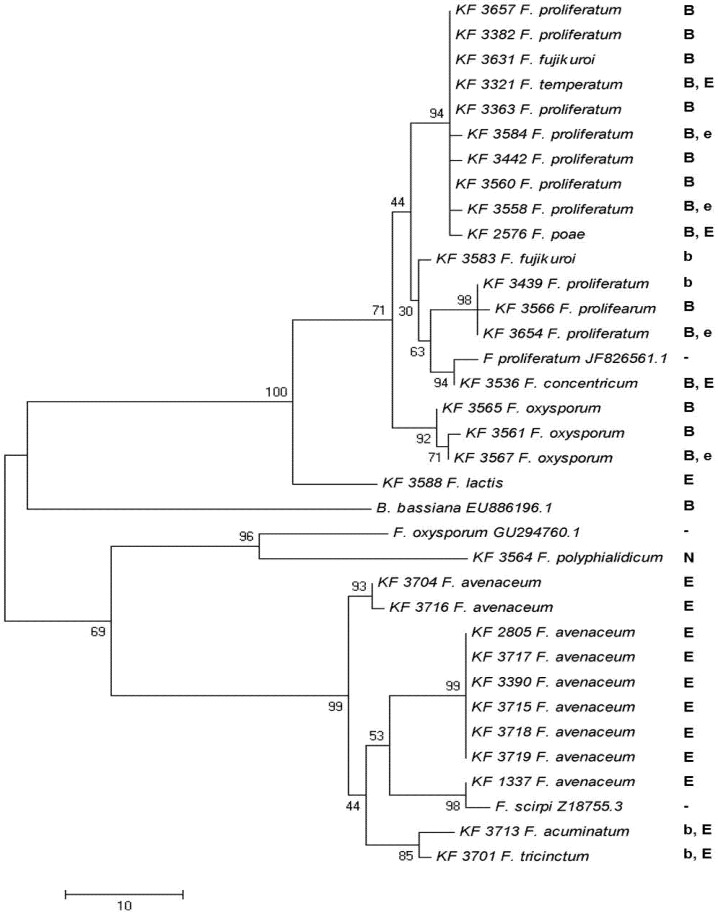
The most parsimonious tree created for a partial enniatin synthase (*esyn1*) gene sequence obtained with Esyn1/Esyn2 or ES_Bea_F/ES_Bea_R primers from 31 strains of eleven *Fusarium* species. GenBank sequences of *esyn1* from *F. scirpi* GenBank ID: Z18755.3, *F. oxysporum* GenBank ID: GU294760.1, *F. proliferatum* GenBank ID: JF8266561.1 and *B. bassiana* GenBank ID: EU886196.1 were included in the analysis. The maximum parsimony approach and bootstrap test were applied (1,000 replicates). “B”, “E”—major—and “b”, “e”—minor—BEA and ENN producers, respectively; N—non-producer.

**Figure 4:**
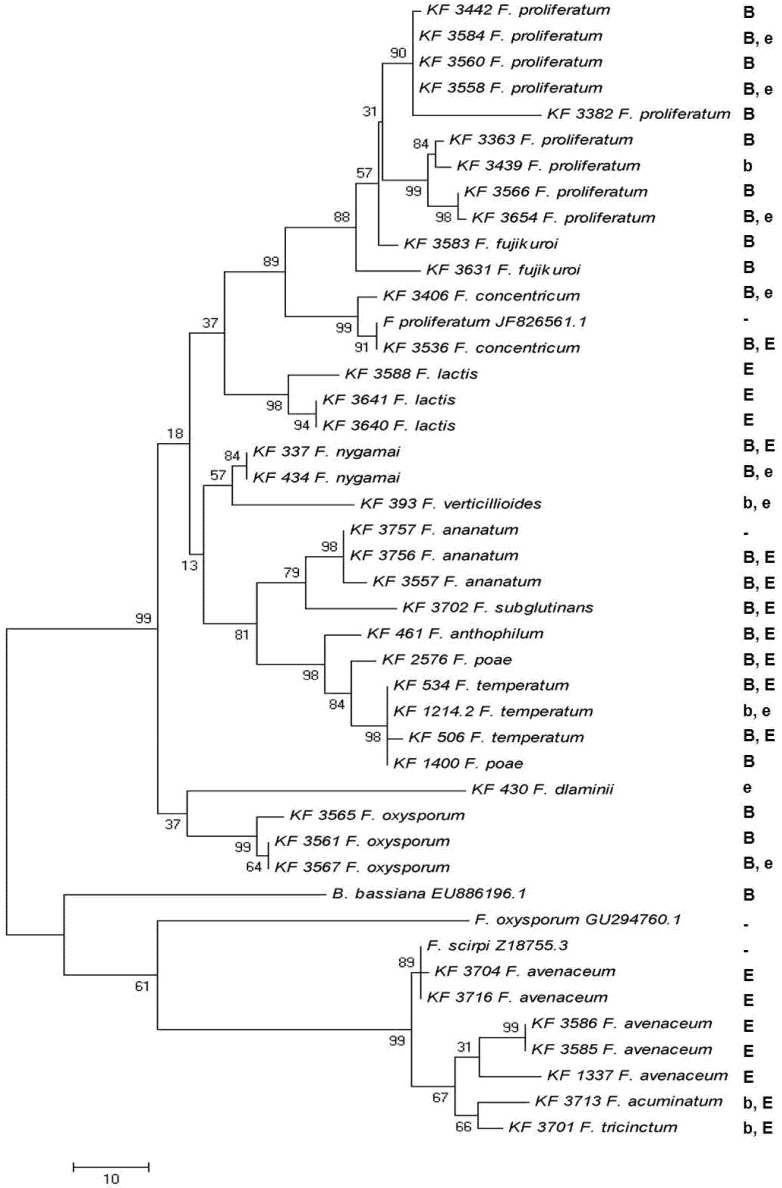
The most parsimonious tree created for a partial enniatin synthase (*esyn1*) gene sequences obtained with beas_1/beas_2 primers from 40 strains of 16 *Fusarium* species. GenBank *esyn1* sequences of *F. scirpi* GenBank ID: Z18755.3, *F. oxysporum* GenBank ID: GU294760.1, *F. proliferatum* GenBank ID: JF8266561.1 and *B. bassiana* GenBank ID: EU886196.1 were included in the analysis. The maximum parsimony approach and bootstrap test were applied (1000 replicates). “B”, “E”—major—and “b”, “e”—minor—BEA and ENN producers, respectively.

PCR products representing two different regions of the enniatin synthase gene, obtained for the majority of the analyzed strains using Esyn1/Esyn2 and beas_1/beas_2 primers, respectively, were sequenced and analyzed. Both regions are located more than 6.5 kbp apart (based on the *F. proliferatum* cluster sequence GenBank ID: JF8266561.1). Regardless of the ENNs/BEA biosynthesis abilities, it was not possible to obtain the marker fragments for some of the strains studied. Namely, *F. ananatum*, *F. anthophilum*, *F. dlaminii, F. nygamai*, *F. subglutinans* and *F. verticillioides* genotypes did not amplify the specific marker fragment using Esyn_1/Esyn_2 and ES_Bea_F/ES_Bea_R primers (Figure 3). Nevertheless, all of the strains amplified the other gene fragment using beas_1/beas_2 primers, and the PCR products were sequenced and analyzed (Figure 4). Moreover, for the *F. nygamai* KF 337 strain, another region of the coding sequence (different from the two covered by the study) was amplified and sequenced. It showed about 80% of nucleotides identical when comparing to *B. bassiana, F. oxysporum* and *F. scirpi* and as much as 89% of identical bases in comparison to *F. proliferatum* sequence (data not shown). For some strain/marker combinations, such as the case of *F. lactis* (KF 3640), *F. polyphialidicum* (KF 3564) and *F. concentricum* (KF 3406) strains, the efficiencies of fluorescent labeling had been significantly lower, which resulted in shorter reads than the remaining sequences aligned. Therefore, these sequences were excluded from the analysis. Finally, no amplification was observed for strains of *F. equiseti*, *F. solani* and *F. sporotrichioides*.

Independent dendrograms were calculated for the enniatin synthase (*esyn1*) fragments obtained with the Esyn/ES_Bea pairs, as well as using the beas_1/2 primers in various genotypes of enniatin- and beauvericin-producers (Figure 3, Figure 4). 

*Fusarium* species, being one of the major pathogens of crop plants worldwide, are considered as producers of some of the most dangerous and harmful mycotoxins present in food and feed. Apart from trichothecenes, fumonisins and zearalenone, cyclic oligopeptides (*i.e.*, beauvericin and enniatins) emerge as a group of toxins commonly present in food products [7], occasionally accumulating in high amounts [12]. 

In the present study, fifty-eight collection strains of 20 *Fusarium* species, representing mainly plant pathogens, but also plant and soil saprophytes, were included. The wide range of hosts and geographical origins proved again the cosmopolitism of the genus. The analysis of the *tef-*1α gene sequences allowed for the discrimination of the species boundaries (Figure 2). This particular gene has been widely and successfully used in phylogenetic studies of *Fusarium* species [45,46,47,48,49,50]; however, the use of the *tef-*1α gene in the studies of a single species genotype variation was limited and often amended by the analysis of different loci [51,52,53]. In the present study, it was possible to differentiate the closely related species, especially belonging to the *G. fujikuroi* species complex and the group of *F. avenaceum/F. acuminatum/F. tricinctum* species. However, as the resolution of the *tef-*1α-based analyses is often limited to the species level, the mycotoxin biosynthetic genes have become versatile and promising tools for analyses aimed at revealing the intraspecific polymorphism [42,43,44,54,55]. 

Therefore, it is justifiable for the enniatin synthase gene (*esyn1*) to have raised significant interest in recent phylogenetic studies of *F. avenaceum* and *F. poae* [12,56]. Both species have been reported to produce ENNs [7,38]. Recently, BEA-producing species have also been identified by cloning and characterization of the respective biosynthetic genes in *B. bassiana* [36] and *F. proliferatum*. Unfortunately, only a few reports are available on the structure of the gene cluster in other BEA producers [37]. 

### 2.5. Toxin Biosynthesis in Relation to the esyn1 Gene Divergence

In the present study, two different regions of the enniatin synthase gene were amplified and analyzed (Figure 3, Figure 4). Both regions are located more than 6.5 kbp apart (based on the *F. proliferatum* cluster sequence GenBank ID: JF8266561.1). The analysis revealed a higher level of polymorphism of *Fusarium* strains than that recorded by the *tef*-1α sequence analysis. However, it was not possible to compare precisely the divergence levels presented by the analyses of both regions. This inconvenience was caused by the significantly lower selectivity of the beas_1/beas_2 primers, which amplified marker fragments from strains of 16 species, while the Esyn1/2 primers were designed and validated only for enniatin-producing *F. avenaceum* and *F. tricinctum* genotypes. Subsequently, the ES_Bea1/2 primers were designed to amplify the corresponding *esyn1* fragment from BEA producers. Eventually, it was possible to obtain the sequences of the strains belonging to 11 species (Figure 3). Since the *esyn1-*based phylogenetic analysis shows clearly “BEA” and “ENN” clades of species and, on the other hand, the majority of the strains produced a mixture of BEA and ENNs, a hypothesis could be drawn that the end-product of the cluster’s activity can possibly undergo some modifications by non-cluster mechanisms. 

## 3. Experimental Section

### 3.1. Fusarium Strains

Fifty-eight *Fusarium* strains were used in the study (Table 1). All strains are stored at the KF *Fusarium* collection (Institute of Plant Genetics, Polish Academy of Sciences, Poznań, Poland). For DNA extraction, seven-day-old cultures grown on potato dextrose agar medium plates were prepared. Harvested mycelia were stored at −20 °C. For toxin biosynthesis analyses, rice cultures of individual strains were used [43].

### 3.2. Mycotoxin Analyses

#### 3.2.1. Apparatus

The chromatographic system used to determine mycotoxin levels consisted of a Waters 2695 high-performance liquid chromatography (HPLC) (Waters, Milford, PA, USA) and a Waters 2996 Photodiode Array Detector with a 150 × 3.9 mm Nova Pak C-18, 4 μm column. Empower™ 1 software was used for data processing (Waters, Milford, PA, USA). 

#### 3.2.2. Chemicals

Enniatins A, A_1_, B, B_1_ and beauvericin standards were purchased with a standard grade certificate from Sigma-Aldrich (Steinheim, Germany). The standard solutions of ENNs (ng μL^−1^) and BEA (ng μL^−1^) were prepared in methanol. Organic solvents (HPLC grade) and all the other chemicals were also purchased from Sigma-Aldrich (Steinheim, Germany). Water for the HPLC mobile phase was purified using a Milli-Q system (Millipore, Bedford, MA, USA).

#### 3.2.3. Extraction and Purification

Culture samples (15 g) of each strain were mixed with 75 mL of extraction mixture—acetonitrile:methanol:water (16:3:1, *v*/*v*/*v*)—then homogenized (homogenizer H500, Pol-Ekoaparatura, Poland) and filtered (Whatman No. 4 filter paper). The extract was centrifuged at 4500*g* for 5 min, and next, the supernatant was evaporated with a Buchi Rotavapor R-210 (Flawil, Switzerland) and then re-dissolved in 2 mL methanol. The final solution was filtered through a 0.45 μm Waters HV membrane filter before injection into the LC-PAD system for analysis. 

#### 3.2.4. HPLC Analysis and Identification

Enniatins and beauvericin, after separation on a 150 × 3.9 mm Nova Pak C-18, 4 μm column, eluted with acetonitrile:water (70:30, *v*/*v*) at a flow rate of 1.0 mL min^−1^, were detected with a Waters 2996 Photodiode Array Detector set at 205 nm. Mycotoxin identification was performed by comparing retention times and UV spectra of purified extracted samples to pure standards. Quantification of mycotoxins was carried out on the basis of a comparison of peak areas with the calibration curve of the standards. All analysis were confirmed with a LC-MS.

#### 3.2.5. Method Validation and Recovery Experiment

For linearity, six-point (5, 10, 20, 40, 60, 80 ng g^−1^) calibration curves were separately prepared for each mycotoxin (ENNs: A, A_1_, B, B_1_ and BEA), and they were obtained using the linear least squares regression procedure of peak area *versus* concentration. 

The recovery experiment was performed on mycotoxin-free rice samples, spiked with three different levels of each mycotoxin separately at a concentration of 5, 20, 60 ng g^−1^. Then, samples were subjected to the procedure, as described in Section 3.2.3. On the basis of these experiments, recovery rates and standard deviations were calculated. 

### 3.3. DNA Extraction*,* PCR Primers*,* Cycling Profiles and DNA Sequencing

Genomic DNAs of all isolates were extracted using a hexadecyltrimethylammonium bromide (CTAB) method, described previously [57]. Primer sequences are given in Table 5. A highly variable fragment of the translation elongation factor 1α (*tef-*1α) was amplified and sequenced using a Ef728M and Tef1R primer pair, validated successfully on *Fusarium* material during previous studies [42,43,44]. The enniatin synthase gene, *esyn1,* was partially amplified using the Esyn_1/Esyn_2 primers designed on the basis of GenBank ID: Z18755.3 sequence from *F. scirpi* [12]. However, it was possible to obtain the marker fragment from only a few BEA-producing strains belonging to *F. nygamai* and *F. proliferatum* (data not shown). Based on the sequence alignment of enniatin and cyclic peptide synthase genes from *F. scirpi, F. oxysporum* (GenBank ID: GU294760.1), *Beauveria bassiana* (GenBank ID: EU886196.1) and several in-house-read sequences, a primer pair was designed to amplify the gene fragment corresponding to the one amplified with Esyn1/Esyn2 primers, both from enniatin and beauvericin-producing species: ES_BeaFand ES_BeaR. Additionally, a pair of degenerated primers were used to amplify the different part of the gene from the studied strains of various *Fusarium* species: beas_1 and beas_2 (Table 5). 

**Table 5:** PCR primers used in the study.

Primer	5'–3' sequence	Amplicon size (bp)	Reference
Ef728M	CATCGAGAAGTTCGAGAAGG	~600	[42,43,44]
Tef1R	GCCATCCTTGGAGATACCAGC		
Esyn_1	GCCGTTGGCGAGCTGGTCAT	995	[12]
Esyn_2	GCAAAGCACGCGTCAACGCA		
ES_BeaF	TCTACAGAACWGGHGAYCTTGC	~750	This study
ES_BeaR	CCYCGCATGCGSACRGCGWARGG		
beas_1	TKGARCAGCGBCAYGAGACM	495	[44]
beas_2	GGWCGRGGGAARTCRGTDGG		

The PCRs were done in 25 μL volumes using PTC-200 and C-1000 thermal cyclers (Bio-Rad, Hercules, CA, USA). Each reaction tube contained 1 unit of Platinum HotStart Taq DNA polymerase (Invitrogen, Carlsbad, CA, USA), 2.5 μL of 10× PCR buffer, 12.5 pmol of forward/reverse primers, 2.5 mM of each dNTP and about 10–20 ng of fungal DNA. PCR parameters were as described: 15 min at 95 °C, 35 cycles of (30–60 s at 94 °C, 30–60 s at 58–64 °C, 1–2 min at 72 °C) and 10 min at 72 °C. Amplicons were electrophoresed in 1.5% agarose gels (Invitrogen, Carlsbad, CA, USA) with ethidium bromide. 

For sequence analysis PCR-amplified DNA fragments were purified with exonuclease I (Epicentre, Madison, WI, USA) and shrimp alkaline phosphatase (Promega, Madison, WI, USA) using the following program: 30 min at 37 °C and 15 min at 80 °C. Both strands were labeled using a BigDyeTerminator 3.1 kit (Applied Biosystems, Foster City, CA, USA), according to Błaszczyk *et al.* [58], and precipitated with ethanol. Sequence reading was performed using Applied Biosystems equipment.

### 3.4. Sequence Analysis and Phylogeny Reconstruction

The sequences of the PCR products were initially aligned with the ClustalW algorithm. Phylogenetic relationships were reconstructed with a MEGA4 software package [59] using the maximum parsimony approach (closest neighbor interchange heuristics). No gap-containing positions were considered in phylogeny analysis. All reconstructions were tested by bootstrapping with 1000 replicates. 

## 4. Conclusions

The phylogenetic relationships revealed on the basis of the constitutively expressed *tef*-1α gene were generally confirmed by the analysis of the *esyn1* gene being involved in the secondary metabolism of *Fusarium* species, with only minor exceptions. Based on both *esyn1* sequence alignments, the strains of *F. poae* were clustered into a group of *F. temperatum*, *F. fujikuroi*, and *F. proliferatum* strains, which formed a strongly supported clade. Both regions analyzed have shown a similar pattern (Figure 3, Figure 4). This could imply a different evolutionary fate of this cluster (or at least the part containing the *esyn1* gene) for *F. poae* than for other species. Similarly, *F. temperatum* positioning differs slightly from the one based on the *tef*-1α sequences. Additional analyses based on different parts of the cluster and, perhaps, also, different genomic regions seem to be necessary to explain this question fully.

Apart from being less stringent, the region amplified using the beas_1/2 primers was also able to reveal a higher level of sequence divergence among the strains analyzed (Figure 4). It could mean that the distal part of the gene is less conserved than the region adjacent to the gene’s beginning. This statement, however, needs to be verified. 

Finally, it was possible to compare the homological sequences from BEA/ENNs producers, as well as from non-producer (*F. polyphialidicum*). This finding, along with the separate clustering of *F. avenaceum* strains, producing mainly ENNs, can implicate the potential use of the BEA/ENN biosynthetic cluster in evolutionary studies of *Fusaria* and other fungal genera. 
